# Dose-dependent and tissue-specific adverse effects of exogenous glucocorticoids: insights for optimizing clinical practice

**DOI:** 10.1007/s40618-025-02637-x

**Published:** 2025-07-07

**Authors:** Riccardo Pofi, Nantia Othonos, Thomas Marjot, Ilaria Bonaventura, Amy Barrett, Sarah White, Hamish Miller, Tom Potter, Maíra Bailey, Richard Eastell, Fatma Gossiel, Conor Woods, Jonathan M. Hazlehurst, Leanne Hodson, Jeremy W. Tomlinson

**Affiliations:** 1 Oxford Centre for Diabetes, Endocrinology and Metabolism, NIHR Oxford Biomedical Research Centre, University of Oxford, Churchill Hospital, Oxford, OX3 7LJ UK; 2 Translational Gastroenterology and Liver Unit (TGLU), Nuffield Department of Medicine, John Radcliffe Hospital, University of Oxford, Oxford, UK; 3 Department of Experimental Medicine, Sapienza University of Rome, Viale Regina Elena, 324, Rome, 00161 Italy; 4 Department of Medicine IV, LMU University Hospital, LMU Munich, Munich, Germany; 5 Barts Liver Centre, Blizard Institute, QMUL, London, UK; 6 Department of Biological and Medical Sciences, Oxford Brookes University, Oxford, UK; 7 Mellanby Centre for Musculoskeletal Research, Division of Clinical Medicine, Faculty of Medicine, Dentistry & Health, University of Sheffield, Sheffield, SR10 2RX UK; 8 Department of Diabetes & Endocrinology, St James Hospital, Dublin, Ireland; 9 Institute of Applied Health Research, University of Birmingham, Edgbaston, Birmingham, UK

**Keywords:** Side effects, Diabetes, Glucose metabolism, Bone health, Prednisolone

## Abstract

**Purpose:**

There is limited data on dose-specific metabolic effects of exogenous glucocorticoids (GC) doses. This study aimed to investigate the differential tissue-specific and dose-dependent effects of low-to-intermediate prednisolone doses on insulin sensitivity and bone metabolism in healthy individuals.

**Methods:**

We performed a post-hoc pooled analysis of three independent clinical trials, each administering one week of daily prednisolone at 10 mg, 15 mg, or 20 mg, in a total of 30 different healthy male volunteers (aged 18–65; BMI 20–35 kg/m²; normal kidney function). Outcome measures included: changes in liver (endogenous glucose production-EGP, β-hydroxybutyrate-OHB), muscle (M/I value, Glucose disposal-Gd) and adipose tissue (NEFA, glycerol) insulin sensitivity assessed across a hyperinsulinemic-euglycemic clamp. Bone turnover was evaluated through osteocalcin levels.

**Results:**

Prednisolone 10 mg had minimal impact on metabolic parameters. 15 mg and 20 mg caused similar reductions (no dose effects) in liver (time effect *p* < 0.05 for EGP and OHB) and skeletal muscle (time effect *p* < 0.05 for M/I and Gd) insulin sensitivity. However, detrimental effects on adipose tissue were dose dependent (dose*time interaction *p* < 0.05 for NEFA and glycerol). Osteocalcin levels decreased similarly after both 15 mg and 20 mg of prednisolone (*p* = 0.199).

**Conclusions:**

Prednisolone-induced insulin resistance exhibits tissue-specific and dose-dependent effects. While 15 mg daily for 7 days appears to induce clinically relevant metabolic changes in this population, the dose-dependent effects observed in adipose tissue suggest tissue-specific variability in response. These findings highlight the importance of dose selection in GC therapy, particularly in individuals predisposed to metabolic complications, though further studies are needed in populations with underlying metabolic disorders.

**Supplementary Information:**

The online version contains supplementary material available at 10.1007/s40618-025-02637-x.

## Introduction

Currently, between 1% and 3% of the global population is prescribed glucocorticoids (GCs) [1–5] and GC use continues to rise [6, 7]. Since their initial application in treating rheumatoid arthritis in the late 1940 s, the immunosuppressive and anti-inflammatory properties of GCs have established them as cornerstones in the treatment of many non-endocrine diseases [8]. Additionally, synthetic GCs are crucial for managing endocrine disorders such as adrenal insufficiency and congenital adrenal hyperplasia in which the aim is to replace GC at physiological doses [9–11]. While the clinical efficacy of GC is well-established, their use is associated with considerable adverse effects [1, 12–14]. Exposure to supraphysiological doses of exogenous GCs, irrespective of formulation, can precipitate severe metabolic, musculoskeletal, and cardiovascular disorders, as well as adrenal suppression and increased risks of infection and mortality. Together, these effects contribute to exogenous or iatrogenic Cushing’s syndrome (CS), which represents the most common form of CS [15–17]. Among the most well-established adverse effects of hypercortisolism are the impairment of glucose metabolism [18] and the GC-induced osteoporosis [19]. The mechanisms underlying GC-induced insulin resistance (IR) and diabetes (DM) in patients with CS involve a combination of pathophysiological pathways [20]. Chronic hypercortisolism exerts direct effects on key peripheral tissues involved in glucose homeostasis, including the liver, skeletal muscle, and adipose tissue [21]. It induces gluconeogenic enzymes in the liver and indirectly promotes gluconeogenesis by stimulating lipolysis and proteolysis, which provide substrates [18]. In bone, GCs increase osteocyte apoptosis, suppress osteoblast activity, and enhance osteoclast-mediated bone resorption. Consequently, bone formation is reduced, especially in trabecular regions, with a corresponding increase in endocortical resorption, both hallmark findings in GC-induced osteoporosis [22]. Although the link between GCs, glucose metabolism and bone impairment has been recognized for over 50 years, significant questions remain concerning the timing and dose thresholds at which synthetic GCs’ adverse metabolic effects become clinically harmful.

Historically, the risk of GC-induced side effects has been considered a function of both dose and duration, with therapeutic benefits increasing in parallel with adverse outcomes [23–25]. Most studies on GC side effects have focused on high doses (> 30 mg/day prednisolone equivalent), but the association of dose and duration with adverse risk is not yet precisely defined. Evidence from several randomized controlled trials suggests that adverse effects at low doses (< 10 mg/day) are generally modest and often not significantly different from placebo [26–28]. However, comprehensive adverse event data in these studies remain limited, and the assumption of low-dose safety does not apply uniformly to all GC-related side effects [28–30]. Importantly, many patients on long-term GC therapy typically receive low-to-intermediate maintenance doses following an initial induction phase [5], but the precise extent to which these doses cause undesired effects remains unclear.

This study seeks to address this critical knowledge gap by investigating and comparing the effects of three commonly prescribed low-to-intermediate therapeutic doses of prednisolone on glucose metabolism and insulin sensitivity across the liver, skeletal muscle, and adipose tissue, as well as their impact on bone health, in healthy subjects.

## Materials and methods

### Study design

We conducted a post-hoc pooled analysis of three independent clinical trials, each evaluating different doses of prednisolone given for one week to different healthy subjects: the FIND-IT study (FI; prednisolone 10 mg/day for one week, reference 15/WA/0071), the PUSH-UP study (PU; prednisolone 15 mg/day for one week, reference 18/SC/0038), and the TICSI study (TI; prednisolone 20 mg/day for one week, reference 08/H0606/107). The results of the FIND-IT [31] and TICSI [32] trials have been previously published. The PUSH-UP trial is presented here for the first time. We aim to compare the effects of these three different doses of oral prednisolone on liver, skeletal muscle, and adipose tissue insulin resistance, as well as on bone metabolism. All studies included an identical investigative protocol with the exception of the administered prednisolone dose. Skeletal muscle insulin resistance was evaluated through the steady-state glucose infusion rate (M, in mg/kg/min) divided by the corresponding plasma insulin concentration (M/I-value) and glucose disposal (Gd), liver insulin resistance through endogenous glucose production (EGP) and β-hydroxybutyrate (OHB), while adipose tissue insulin resistance was evaluated through the circulating levels of non-esterified fatty acid (NEFA) and glycerol. These outcomes were collected from the low-dose phase (20 mU/m^2^/min) of the two-step *hyperinsulinemic-euglycemic* clamp performed in each trial. Only the low dose assessments were included in the analysis because the supraphysiological amount of insulin administered during the high dose phase (100 mU/m^2^/min) masked any significant GC impact. Bone health was assessed through circulating osteocalcin levels; however, osteocalcin data were only available for the PU and TI studies. Additionally, we collected data on routine biochemistry, endocrine and lipid profiles from each trial and compared the changes in metabolic and clinical variables across the three different doses (10, 15 and 20 mg) of prednisolone. The change in any outcome was calculated as: Δ = post-treatment *minus* pre-treatment assessment. All three trials shared identical inclusion criteria, enrolling male healthy volunteers aged 18–65 years with a BMI of 20–35 kg/m^2^ and normal eGFR (> 60 mL/min/1.73 m^2^). Key exclusion criteria included a history of Type 1 or Type 2 DM, hypertension, recent use of GC therapy (within the last six months), medications affecting GC metabolism, and abnormal liver function tests. Full inclusion and exclusion criteria for each trial are detailed in published reports [31, 32]. Written informed consent was obtained from all subjects.

### Procedures

All the subjects performed identical procedures and all attended the Clinical Research Unit, Churchill Hospital (Oxford, UK) at 8:00 AM after an overnight fast (from 24:00 h). Fasting blood samples were taken for biochemistry, endocrine, lipid profiles. After confirmed eligibility, participants returned to the CRU the following day having fasted from 24.00 h the previous night. At 08.00 h, a low-dose hyperinsulinemic euglycemic clamp was started.

### Low-dose hyperinsulinemic-euglycemic clamp

On commencement of the hyperinsulinemic-euglycaemic clamp, a bolus of U-^13^C-glucose (Cambridge Isotope Laboratories, Andover, USA) was administered (2 mg/kg over 1 min followed by a continuous infusion in 0.9% saline (20 µg/kg/min). Blood glucose was monitored at 15-minute intervals during the initial 120 min (t = 0–120 min) basal phase. At t = 120 min, an insulin infusion (Actrapid; Novo Nordisk) was infused at 20 mU/m^2^/min (low-dose) alongside an infusion of 20% dextrose supplemented with U-^13^C-glucose enriched to 4%; blood glucose levels were monitored at 5-minute intervals (t = 120–240 min). Blood samples were taken at 2 time points in the last 30 min of each phase (basal and low-insulin) for steady state measurements of insulin, whole body glucose turnover (Ra glucose, Glucose disposal) and EGP. Glucose disposal (Gd) rates was calculated using a modified version of the Steele equations [33, 34]. Participants were then prescribed with prednisolone 10 mg, 15 mg or 20 mg once daily (OD) in the morning (7–9 AM) for one week, as assigned based on their specific study group (FI, PU or TI study, respectively). The participants returned to the research facility on the last day of administration and all investigations were repeated.

### Clinical relevance of measured parameters

We performed a comprehensive evaluation of tissue-specific insulin sensitivity, enabling an in-depth understanding of how prednisolone influences metabolic regulation across liver, muscle, and adipose tissues. We evaluated multiple clinically relevant metabolic parameters. M/I value were derived from hyperinsulinemic-euglycemic clamps, reflecting whole-body insulin sensitivity with a primary focus on skeletal muscle response to insulin. Lower M/I values indicate reduced glucose uptake in muscle, commonly observed in insulin-resistant states. Glucose disposal rate (Gd) during the clamp measures the total uptake of glucose by muscle and peripheral tissues, providing a broader insight into insulin responsiveness. We measured EGP to assess hepatic insulin sensitivity. Insulin normally suppresses EGP by inhibiting gluconeogenesis and glycogenolysis. Elevated EGP during insulin infusion suggests hepatic insulin resistance. Glycerol and NEFA were assessed to gauge adipose tissue insulin sensitivity. Elevated circulating glycerol and NEFA levels indicate increased lipolysis, which is typically suppressed by insulin. Higher levels of these markers reflect impaired insulin-mediated suppression of lipolysis, meaning adipose insulin resistance and contribute to metabolic complications by increasing substrate availability for hepatic glucose production. Lastly, OHB was measured and considered as a proxy for hepatic insulin sensitivity. Within the liver, OHB is produced through β-oxidation of free fatty acid. Under normal conditions, insulin promotes lipogenesis (e.g. de novo lipogenesis) and suppresses β-oxidation. Thus, elevated OHB levels, particularly in the presence of insulin, indicate impaired hepatic insulin signalling and reduced insulin sensitivity) [35]. We measured osteocalcin to capture the early impact of prednisolone on bone health. Osteocalcin is a bone-specific protein synthesized by osteoblasts, which plays a critical role in bone formation and mineralization [36]. Reduced osteocalcin levels have been associated with increased bone resorption, making it a valuable indicator of skeletal health.

### Biochemical and stable isotope analysis

Cholesterol, liver biochemistry and plasma glucose were measured using standard laboratory methods (Roche Modular system, Roche Ltd, Lewes, UK). Insulin and osteocalcin were measured using a commercially available colorimetric ELISAs (Insulin: Mercodia, Uppsala, Sweden; Osteocalcin: Thermofisher, Frederick, USA). Serum and plasma samples were analysed using standard laboratory methods as previously described [31]. Plasma enrichment of U-13 C-glucose was measured using gas chromatography-mass spectrometry (model 5973; Agilent Technologies, Cheshire, United Kingdom). Glycerol, NEFA and OHB were measured using commercially available kits on an ILAB600/ILAB650 clinical analyser (Instrumentation Laboratory UK, Warrington, UK).

### Statistical analysis

Data are presented as mean ± SE or median [95% CI], depending on the distribution of data, which was assessed using the Shapiro–Wilk test. Linearity was verified through visual inspection of scatterplots. To evaluate the effects of treatment over time and across dose groups, we used a two-way repeated measures ANOVA, with time (baseline vs. post-treatment) as the within-subject factor and dose group (10 mg, 15 mg, 20 mg) as the between-subject factor. This model allowed us to estimate the effect of treatment within each group and simultaneously compare the magnitude (delta) of treatment-related changes across dose groups (described as dose*time interaction throughout the results), while appropriately accounting for baseline imbalances between groups. The F statistic is reported as part of the ANOVA results, indicating the ratio of between-group variance to within-group variance. A significant F value (*p* < 0.05) suggests that at least one group mean differs from the others. Partial eta squared (η²) was calculated to estimate effect sizes and quantify the proportion of variance explained by treatment and dose. Effect sizes were interpreted using conventional thresholds (η² ~0.01: small; >0.06: medium; >0.14: large), providing additional context for the magnitude of observed changes beyond p-values alone [37]. Post-hoc tests were performed for pairwise comparisons to identify specific differences, with Bonferroni correction to adjust for multiple testing. Data are reported as mean and 95% confidence intervals unless otherwise stated, and the threshold for statistical significance was set at *p* < 0.05. Statistical analyses were performed using SPSS (version 24, Chicago, IL, USA) and GraphPad Prism 7.0 software package (GraphPad Software, Inc. La Jolla, CA, USA).

## Results

Thirty healthy male volunteers were included in the analysis: five subjects from the FIND-IT study randomized to prednisolone 10 mg, ten subjects from the PUSH-UP study prescribed with prednisolone 15 mg and fifteen from the TICSI study randomized to prednisolone 20 mg. The three groups were balanced for baseline characteristics (Table 1). After one week of prednisolone (10 mg, 15 mg or 20 mg), no differences were found in electrolytes, lipid profile, kidney and liver function. Similarly, systolic and diastolic blood pressure also remained stable in all treatment groups at the end of the study (data not shown).

**Table 1:** Baseline characteristics of the 30 healthy volunteers enrolled to the FIND-IT (10 mg of prednisolone), PUSH-UP (15 mg of prednisolone) and TICSI (20 mg of prednisolone) trials

	FIND-IT (31) (*n* = 5)	PUSH-UP (*n* = 10)	TICSI (32) (*n* = 15)
Age, y	47 [37;49]	48 [45;52]	38 [25;50]
Weight, Kg	88.7 ± 7.5	83.6 ± 6.6	79.4 ± 10.1
BMI, kg/m2	27.8 ± 3.2	25.6 ± 2.0	24.9 ± 2.4
SBP, mm Hg	138 ± 3*	129 ± 16	128 ± 9
DBP, mm Hg	88 ± 6	80 ± 8	79 ± 7
HbA1c, mmol/mol	34 ± 3	34 ± 3	34 ± 3
Fasting glucose, mmol/L	4.7 ± 0.6	4.5 ± 0.4	4.8 ± 0.5
Fasting Insulin, mmol/L	3.2 ± 2.1	4.2 ± 1.7	3.2 ± 2.3
HOMA-IR	0.7 ± 0.5	0.8 ± 0.3	0.7 ± 0.5
AST, IU/L	21 ± 5	20 ± 4	22 ± 7
Total Cholesterol, mmol/L	5.3 ± 1.0	5.0 ± 0.9	4.6 ± 0.9
eGFR, mL/min/1.73m^2^	90 [82;90]	90 [82;90]	90 [87;90]

AST, aspartate aminotransferase; BMI, body mass index; DBP, diastolic blood pressure; SBP, systolic blood pressure, eGFR, estimated glomerular filtration rate. Data are expressed as mean ± SD or median [IQR] according to data distribution

*p < 0.05 vs. TICSI

### Common routine metabolic assessments

**Fig. 1:**
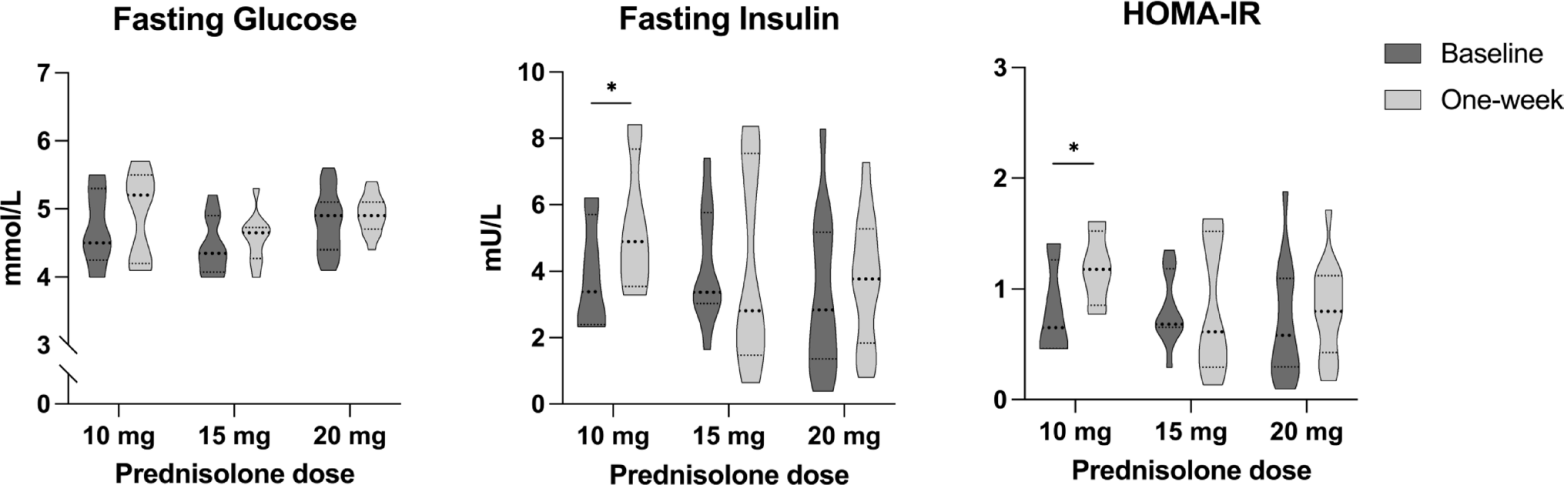
Summary of changes in common routine metabolic assessments in healthy volunteers across the three studies (FIND-IT, PUSH-UP and TICSI) before and one-week after prednisolone treatment. Legend: *p **<** 0.05

There was no effect of any prednisolone dose on fasting glucose levels (ΔGlucose_10mg_ 0.2 mmol/L, 95%CI −0.2 to 0.6, *p* = 0.299; ΔGlucose_15mg_ 0.1 mmol/L, 95%CI −0.1 to 0.4, *p* = 0.377; ΔGlucose_20mg_ 0.1 mmol/L, 95%CI −0.1 to 0.4, *p* = 0.380). Insulin levels (ΔInsulin_10mg_ 2.9 mU/L, 95%CI 0.7 to 5.2, *p* = 0.02) and HOMA-IR (ΔHOMA-IR_10mg_ 0.50, 95%CI 0.07 to 1.05, *p* = 0.026) increased modestly in subjects taking 10 mg of prednisolone, while there were no changes in the 15 mg (ΔInsulin_15mg_ −0.1 mU/L, 95%CI −1.5 to 1.4, *p* = 0.935; ΔHOMA-IR_15mg_ 0.01, 95%CI −0.29 to 0.32, *p* = 0.938) and 20 mg (ΔInsulin_20mg_ 0.5 mU/L, 95%CI −0.7 to 1.7, *p* = 0.411; ΔHOMA-IR_20mg_ 0.12, 95%CI −0.14 to 0.37, *p* = 0.347) groups, providing a non-significant dose*time interaction (*p* = 0.09 and *p* = 0.16, respectively) (Fig. 1).

### Low-dose hyperinsulinemic-euglycemic clamp

Table 2 summarizes the changes in insulin sensitivity across skeletal muscle, liver and adipose tissue as measured across the low-dose hyperinsulinemic-euglycemic clamp.

**Table 2:** Summary of glucose metabolism parameters from low-dose hyperinsulinaemic-euglycaemic clamp at baseline and one-week after prednisolone treatment in healthy volunteers participating the FIND-IT (10 mg), PUSH-UP (15 mg) and TICSI (20 mg) studies

	Δ_10mg_	*p*	Δ_15mg_	*p*	Δ_20mg_	*p*	Time*Dose interaction *p*
*Skeletal muscle*							
M/I value (mg/Kg•min per mUI/L)	−6.2[−13.1;0.69]	0.076	−7.1[−12.4;−1.7]	**0.011**	−10.6[−15.0;−6.2]	**< 0.001**	0.433
Glucose disposal (mg/Kg•min)	−0.8[−2.2;0.6]	0.273	−1.7[−2.8;−0.7]	**0.002**	−1.6[−2.4;−0.7]	**< 0.001**	0.514
*Liver*							
EGP (mg/Kg•min)	−0.1[−0.5;0.3]	0.581	0.4[0.1;0.80]	**0.020**	0.4[0.1;0.6]	**0.004**	0.090
OHB (µmol/L)	0.83[−3.2;4.8]	0.673	4.8[1.5;8.1]	**0.006**	4.7[2.0;7.5]	**0.001**	0.673
*Adipose tissue*							
NEFA (µmol/L)	−4.5[58.3;49.4]	0.865	59.1[20.3;97.9]	**0.004**	75.4[41.4;109.4]	**< 0.001**	**0.050**
Glycerol (µmol/L)	1.75[−0.1;3.52]	0.053	5.6[4.2;7.0]	**< 0.001**	4.7[3.6;5.8]	**< 0.001**	**0.005**
*Bone*							
Osteocalcin (ng/mL)	/	/	−2.77[−4.41;−1.14]	**0.002**	−4.10[−5.36;−2.83]	**< 0.001**	0.199

Data are presented as mean [95%CI]; significant *p* value are highlighted in bold. Results refer to the two-way repeated measures ANOVA analysis. Δ = post-treatment *minus* baseline assessment; M/I value: steady-state glucose infusion rate divided by the corresponding plasma insulin concentration; EGP: endogenous glucose production; OHB: β-hydroxybutyrate; NEFA: non-esterified fatty acid

### Liver and skeletal muscle insulin sensitivity

**Fig. 2:**
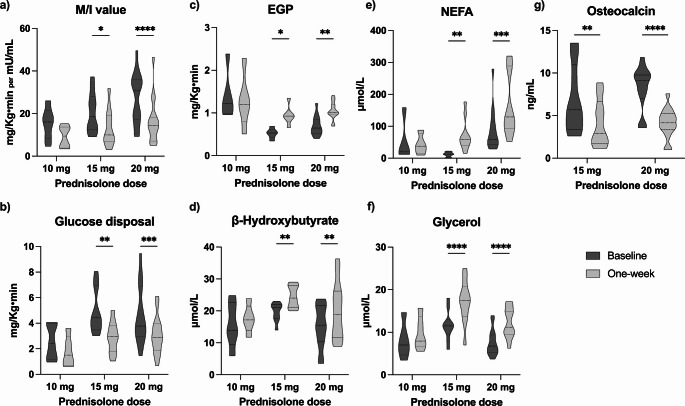
Summary of changes in M/I and glucose disposal (skeletal muscle, **a** and **b**), endogenous glucose production rate (EGP) and β-hydroxybutyrate (liver, **c** and **d**) and non-esterified fatty acids (NEFA) and glycerol (adipose, **e** and **f**) across the low-dose hyperinsulinemic-euglycemic clamp in healthy volunteers enrolled in the three studies (FIND-IT, PUSH-UP and TICSI), before and one-week after prednisolone treatment. (**g**) osteocalcin levels in healthy volunteers enrolled in the PUSH-UP and TICSI studies, before and one-week after prednisolone treatment. Legend: *p **<** 0.05; **p **<** 0.01; ***p **<** 0.001; ****p **<** 0.0001

The M/I value exhibited a robust effect of *time* (F = 25.02, η²=0.47, *p* < 0.001), indicating an notable decline from baseline to one-week post-treatment. Significant reductions were noted in the 15 mg (−7.1 mg/kg•min, 95% CI −12.4 to −1.7, *p* = 0.011) and 20 mg (−10.6 mg/kg•min, 95% CI −15.0 to −6.2, *p* < 0.001) groups, with only borderline effects at the 10 mg dose (−6.2 mg/kg•min, 95% CI −13.1 to 0.7, *p* = 0.076). The dose*time interaction was not significant (*p* = 0.433), emphasizing a uniform decline across the 15 mg and 20 mg doses. (Fig. 2).

Analysis of stable isotope calculated Gd revealed a meaningful *time* effect (F = 18.57, η²=0.42, *p* < 0.001), suggesting a reduction post-treatment. Post-hoc tests confirmed decreases in *Gd* for both the 15 mg (−1.7 mg/kg•min, 95% CI −2.8 to −0.7, *p* = 0.002) and 20 mg doses (−1.6 mg/kg•min, 95% CI −2.4 to −0.7, *p* < 0.001), while the 10 mg dose showed no significant change (*p* = 0.273). The lack of dose*time interaction (*p* = 0.514) again support a consistent reduction of insulin sensitivity across the higher doses.

The analysis of EGP was affected by both *dose* (F = 9.54, η²=0.46, *p* = 0.001) and *time* (F = 5.94, η²=0.21, *p* = 0.020), reflecting a moderate-to-large treatment-related increase. Post-hoc comparisons highlighted a significant increase in EGP for the 15 mg dose (0.4 mg/kg•min, 95% CI 0.1 to 0.8, *p* = 0.020) and 20 mg dose (0.4 mg/kg•min, 95% CI 0.1 to 0.6, *p* = 0.004), while the 10 mg dose remained unaffected (*p* = 0.581). However, there was only a trend on dose*time interaction (*p* = 0.090), indicating the increase in EGP did not significantly differ across higher doses.

Also OHB levels increased over *time* (F = 13.18, η²=0.34, *p* = 0.001) with change observed at both the 15 mg dose (4.8 mmol/L, 95% CI 1.5 to 8.1, *p* = 0.006) and the 20 mg dose (4.7 mmol/L, 95% CI 2.0 to 7.5, *p* = 0.001), and no change at the 10 mg dose (*p* = 0.673). As for M/I value and EGP, the lack of dose*time interaction (*p* = 0.226) suggested a consistent increase in β-hydroxybutyrate levels across doses over time.

### Adipose tissue insulin sensitivity

Overall, significant changes were observed, notably after the 15 mg and 20 mg doses, with incremental effects across doses, indicating a dose-dependent reduction in adipose tissue insulin sensitivity.

The two-way ANOVA for NEFA levels showed a strong main effect of both *dose* (F = 7.61, η²=0.37, *p* = 0.002) and *time* (F = 12.89, η²=0.34, *p* = 0.001), indicating substantial increase across treatment groups. NEFA rose significantly at the 15 mg (59.1 µmol/L, 95% CI 20.3 to 97.9, *p* = 0.004) and the 20 mg doses (75.4 µmol/L, 95% CI 41.4 to 109.4, *p* < 0.001), with no change at the 10 mg dose (*p* = 0.865). The dose*time interaction was significant (η²=0.21, *p* = 0.050), suggesting that the variation in NEFA increased with increasing prednisolone dose after 15 mg of prednisolone.

Glycerol showed very pronounced effects, with a large effect of *dose* (F = 5.99, η²=0.30, *p* = 0.007) and strong effect of *time* (F = 96.63, η²=0.77, *p* < 0.001). Post-hoc analysis showed increase in glycerol levels from baseline to one week at the 15 mg dose (5.6 µmol/L, 95% CI 4.2 to 7.0, *p* < 0.001) and the 20 mg dose (4.7 µmol/L, 95% CI 3.6 to 5.8, *p* < 0.001), while only borderline change was observed in the 10 mg dose group (1.7 µmol/L, 95% CI −0.1 to 3.5, *p* = 0.053). As per NEFA, the significant dose*time interaction (F = 6.42, η²=0.31, *p* = 0.005) further supported a dose-amplified response on glycerol release.

### The effects of prednisolone on bone

To assess the impact of prednisolone on bone, we measured osteocalcin levels pre- and post-treatment. Both 15 mg and 20 mg doses of prednisolone were associated with a significant reduction in osteocalcin, with the effect more pronounced at the higher dose (Δ_15mg_ −2.77 ng/mL, 95% CI −4.41 to −1.14, *p* = 0.002; Δ_20mg_ −4.10 ng/mL, 95% CI −5.36 to −2.83, *p* < 0.001). However, when comparing the median reductions between the two doses, no difference was observed (*p* = 0.199), suggesting that both doses impair bone turnover similarly within the observed sample. Unfortunately, osteocalcin levels were not available in patients belonging to the 10 mg group.

## Discussion

This study provides a retrospective comparison of the differential tissue-specific and dose-dependent adverse effects of three commonly used low-to-intermediate doses of prednisolone using a hyperinsulinemic-euglycemic clamp. Our findings suggest that standard clinical metrics, such as fasting glucose, fasting insulin, and HOMA-IR, may lack sensitivity in detecting prednisolone-induced changes in insulin sensitivity, whereas the low-dose hyperinsulinemic-euglycemic clamp revealed important changes in metabolic and bone phenotype, even at short-term exposure. Specifically, a 10 mg dose of prednisolone exerts only mild effects on insulin sensitivity, while 15 mg and 20 mg doses result in significant, tissue-specific reductions in insulin sensitivity, impacting skeletal muscle, liver, and adipose tissue differently.

Our data suggest that metabolic effects emerge at doses of 15 mg, with little additional detriment seen at 20 mg across this short intervention period, though the magnitude of these effects varied by tissue. Both 15 mg and 20 mg doses reduced skeletal muscle insulin sensitivity, indicated by decreased M/I-values, and glucose disposal, and increased hepatic gluconeogenesis and glycogenolysis, as shown by elevated EGP. Notably, the liver appears resistant to additional impairment of EGP beyond the 15 mg dose, suggesting a potential ceiling effect of prednisolone’s short-term impact on hepatic glucose metabolism in healthy individuals. In contrast, adipose tissue sensitivity to prednisolone appear to decline in a dose-dependent fashion, with progressive increases in circulating NEFA and glycerol observed from dose above 15 mg, suggesting that adipose tissue may be more sensitive to dose escalation than liver or muscle. However, given the small number of participants in the 10 mg group, these findings should be interpreted with caution. The limited sample size reduces the power to detect subtle metabolic effects at this lower dose, and further studies in larger cohorts are needed to confirm whether 10 mg truly represents a threshold below which glucocorticoid-induced metabolic alterations are negligible.

There are few published studies that have used the precision metabolic phenotyping employed in this study to define the metabolic effects of short-term isolated GC treatment. In patients with inflammatory rheumatological disease, a 7- to 10- day of prednisolone treatment (6 mg) results in increased basal EGP, reduced glucose disposal [38], and increased peripheral insulin resistance as measured with Matsuka index [39], suggesting that the liver and skeletal muscles are sensitive to GC-induced insulin resistance, even at low doses, especially in populations predisposed to metabolic dysfunction, such as patients with autoimmune diseases. Our results align with these findings concerning the increased HOMA-IR in patients taking 10 mg of prednisolone. In contrast, in our healthy cohort, hepatic and skeletal muscle insulin resistance was more evident at 15 mg and above, suggesting that baseline metabolic status may influence glucocorticoid sensitivity [40]. Differences in age and underlying conditions likely contribute to these variations, emphasizing the need for individualized risk assessment in clinical practice. Interestingly, another study showed decreased insulin-dependent suppression of EGP healthy in male volunteers with longer (2-week) treatment with 7.5 mg of prednisolone [41], emphasizing the role of treatment duration in GC-induced metabolic dysfunction.

High-dose GC regimens (30–75 mg) are known to exacerbate insulin resistance in liver, muscle, and adipose tissue both in healthy volunteers and in patients with autoimmune diseases [40–42]. Our study fills a critical knowledge gap by focusing on the intermediate doses (10 to 20 mg) that are commonly prescribed long-term. While our findings suggest that 10 mg of prednisolone, a dose slightly above physiological cortisol replacement levels, has modest metabolic effects, escalating to 15 mg or 20 mg induces pronounced insulin resistance across multiple tissues. Although fasting glucose remained stable across doses, we observed increases in insulin and HOMA-IR at 10 mg, indicating early compensatory responses without significant impairment in insulin sensitivity as measured by the clamp [40]. At 15 mg, however, a greater magnitude of metabolic change was observed, with both central (hepatic) and peripheral (skeletal muscle and adipose) insulin sensitivity being markedly impaired. Notably, there were differences in tissue-sensitivity to the prednisolone detrimental effects. While hepatic insulin sensitivity plateaus between 15 mg and 20 mg, adipose tissue sensitivity continues to decline with escalating doses, as evidenced by the progressive increase in NEFA and glycerol levels. These findings align with Van Raalte et al. [41] who noted no significant changes in NEFA after two weeks of 7.5 mg prednisolone, but observed adipose insulin resistance at higher doses. Our data also provide evidence for adipose tissue being less responsive to the effects of GC at doses < 15 mg.

These findings have significant clinical implications regarding hepatic lipid accumulation and the predisposition to liver steatosis. Insulin resistance is a central pathological component of metabolic disorders, including type 2 DM, obesity, and metabolic dysfunction-associated steatotic liver disease (MASLD), now a major global health challenge. MASLD, characterized by hepatic lipid accumulation (steatosis), is a precursor to progressive liver diseases such as fibrosis, cirrhosis, and hepatocellular carcinoma [43]. In insulin-sensitive individuals, insulin suppresses adipocyte lipolysis, enhances hepatic de novo lipogenesis (DNL), and inhibits β-oxidation [44]. However, GC-induced insulin resistance disrupts these mechanisms. In adipose tissue, IR results in sustained release of NEFA into the circulation, leading to hepatic free fatty acid (FFA) overload [45]. In the liver, increased β-oxidation and OHB production may initially act as compensatory mechanisms to manage the FFA influx. However, with persistent and excessive FFA flux, mitochondrial oxidative capacity becomes overwhelmed, leading to a plateau in these compensatory processes [35]. Preclinical evidence suggests that high doses of dexamethasone can suppress hepatic β-oxidation, impair mitochondrial function, and promote lipid storage [46]. This incomplete β-oxidation leads to an imbalance between lipid influx and efflux, favouring hepatic lipid accumulation. While we did not measure intrahepatic lipid content in this study, our findings support the concept of mitochondrial overload and impaired oxidative capacity in the 20 mg dose group. This is reflected in the increased OHB levels observed at the 15 mg dose of prednisolone, which plateaued at the 20 mg dose despite increased circulating NEFA levels.

While our data demonstrate impaired insulin sensitivity, low-dose GC treatment is associated with a lower incidence of diabetes in prospective studies [47], possibly due to reliance on fasting glucose measurements, which does not capture nuanced changes in glucose handlining and insulin sensitivity. Similar to other reports [31, 41, 48–50] our findings suggest that subtle increases in insulin and HOMA-IR observed in low doses may not be clinically meaningful. Variability in magnitude of effects across studies likely reflects differences in treatment duration, dose, and population characteristics. The short, one-week duration of treatment in our healthy cohort enables precise measurement of GC metabolic effects absent of confounding inflammation, yet clinical translation should consider the chronic, often inflammatory, conditions in which GCs are prescribed. Systemic inflammation itself can impair insulin sensitivity, and the metabolic impacts of GCs may thus be magnified in such populations [51].

The impact of prednisolone on bone metabolism, reflected by reductions in osteocalcin levels, also highlights a significant concern. Despite the small number of patients, and the short intervention, both 15 mg and 20 mg doses led to similar declines in osteocalcin. Unfortunately, osteocalcin levels were not measured in the FIND-IT trial, limiting broader validation of these results across varied patient populations. Previous studies have shown that prednisolone suppresses osteocalcin in a dose-dependent manner, with reductions observed at doses of 10 mg and above [52]. However, our findings did not detect a significant difference between 15 mg and 20 mg, which may reflect the short duration of treatment or individual variability in bone metabolism. Further studies are needed to assess whether osteocalcin suppression exhibits a plateau effect at intermediate prednisolone doses.

Our study has some limitations. Firstly, the study was not originally designed for direct dose comparisons, and the sample size, particularly for the 10 mg group, may have limited our ability to detect subtle dose effects. Secondly, while our findings highlight short-term metabolic responses, long-term effects remain uncertain. Finally, given that the study was conducted in healthy male volunteers treated for one week, it is important to emphasize that these findings reflect short-term GC exposure in a metabolically healthy population, and extrapolation to women, elderly individuals, or those with metabolic disorders requires caution as responses may differ in individuals with pre-existing insulin resistance or inflammatory conditions. However, leveraging data from three separate trials with consistent protocols, adds robustness to the findings and provides a broad basis for comparing the differential metabolic impacts of these doses. The hyperinsulinemic-euglycemic clamp, is not feasible for routine clinical practice due to its complexity and need for hospitalization. Nevertheless, its use provides precise, high-sensitivity measurements of tissue-specific insulin sensitivity across liver, muscle, and adipose tissues, distinguishing this work from studies relying solely on fasting glucose or surrogate indices like HOMA-IR or Matsuka index. This technique allows for a nuanced understanding of how low-to-intermediate doses of prednisolone impact metabolic health in a way that clinical markers alone cannot capture. Additionally, by including three different prednisolone doses, our study enhances our understanding of the dose threshold at which GC-induced insulin resistance becomes clinically meaningful. This comprehensive dose-response evaluation, combined with the analysis being undertaken in a healthy cohort, allows us to isolate the direct metabolic effects of prednisolone without confounding variables such as underlying inflammation.

## Conclusions

Our study provide new insights into the glucocorticoid-induced insulin resistance, revealing it as a nuanced, dose-dependent, and tissue-specific phenomenon that may be missed by routine clinical assessments. While one week of low-dose prednisolone treatment (10 mg) exerts mild effects in healthy subjects, a modest increase from 10 mg to either 15 or 20 mg markedly amplifies metabolic disruptions in a dose- and tissue-dependent manner. This distinction is crucial, as published data indicate that individuals with inflammatory conditions may experience adverse metabolic effects at even lower doses, underscoring the importance of individualized dosing. These findings highlight the importance of careful dose selection in glucocorticoid therapy and a tailored approach to dosing, favouring lower doses around 10 mg when clinically feasible, can help minimize adverse metabolic effects. However, doses of 15 mg and above induce widespread insulin resistance across liver, skeletal muscle, and adipose tissue, emphasizing the need for early intervention strategies. Larger, prospectively designed studies are needed to confirm these findings in broader populations, particularly in individuals with pre-existing metabolic disease. The tissue-specific responses observed here, however, reinforce the value of a personalized dosing strategy to mitigate long-term complications. Physicians prescribing glucocorticoids should remain vigilant about potential metabolic and bone disturbances and consider early preventive measures to counteract these effects, optimizing patient outcomes over the course of treatment.

## Electronic supplementary material

Below is the link to the electronic supplementary material.

Supplementary Material 1

## Data Availability

The datasets generated and analysed during the current study are not publicly available but are available from the corresponding author on reasonable request.
