# Supplementary material for: Dose-dependent and tissue-specific adverse effects of exogenous glucocorticoids: insights for optimizing clinical practice
Source: J Endocrinol Invest. 2025 Jul 7;48(9):2067–76. doi: 10.1007/s40618-025-02637-x (PMC12398475; doi:10.1007/s40618-025-02637-x)
Supplement: Supplementary file 1 — Supplementary Material 1 [file 40618_2025_2637_MOESM1_ESM.docx]

**Response to reviewer’ comments for: JENI-D-25-00546**

***“Dose-Dependent and Tissue-Specific Adverse Effects of Exogenous Glucocorticoids: Insights for Optimizing Clinical Practice”***

Dear Professor Pagotto

We would like to thank you and the reviewer for the kind reply, but also for the time and effort spent on reviewing the manuscript. We have read your suggestions, and shared the reviewer’s comments with the co-authors. All of the comments and suggestions have been addressed and incorporated in the revised version, we believe that they have refined and improved the manuscript. Below is a summary of comments and our responses.

| **Reviewer 1 general comment:** | | | |
| --- | --- | --- | --- |
| Thank you very much for asking me to review this interesting and detailed manuscript.  In their paper the authors aimed to investigate the differential tissue-specific and dose-dependent effects of low-to-intermediate prednisolone doses on insulin sensitivity across a hyperinsulinemic-euglycemic clamp and bone health in healthy male individuals. In particular a post-hoc pooled analysis of three independent clinical trials, each administering one week of daily prednisolone at 10 mg, 15 mg, or 20 mg to 5, 10 and 15 subjects respectively, was performed. Changes in liver (endogenous glucose production-EGP, β-hydroxybutyrate-OHB), muscle (M/I value, Glucose disposal-Gd) and adipose tissue (NEFA, glycerol) have been evaluated. The authors concluded that one week of 15 mg and 20 mg caused similar reductions in liver and skeletal muscle insulin sensitivity, whereas, detrimental effects on adipose tissue were dose dependent, suggest tissue-specific variability in response. Osteocalcin levels decreased similarly after both 15 mg and 20 mg of prednisolone.  The study is well written, results are almost clear and properly discussed, limitations included.  Indeed, the study is focused only on male subjects, each group included a limited number of patients and the duration of follow-up is just 1week, limiting the strengths of conclusions and transferability to clinical practice where the use of glucocorticoids is often chronical. | | | |
|  | **Reviewer’ specific comments** | **Author response and changes made** | **Page and line numbers in revised paper where the change can be found** |
| **1** | Have the results of the three independent clinical trials been published jet? It should be specified. | *We thank the reviewer for this important observation. Two of the three clinical trials contributing to the pooled dataset have been previously published. Specifically, data from the five participants treated with 10 mg of prednisolone were derived from the FIND-IT trial (J Clin Endocrinol Metab. doi: 10.1210/clinem/dgaa408), and the results from the 15 participants treated with 20 mg of prednisolone were previously reported as part of the TICSI trial (Nat Commun. doi: 10.1038/s41467-023-36541-w).*  *The present study is the first to report the findings from the third trial, PUSH-UP, which includes 10 participants treated with 15 mg of prednisolone.*  *We have now revised the Methods section to clarify the origin of the dataset and to explicitly state which data are published and which are presented here for the first time.* | Page 6,  lines 107-109 |
| **2** | Statistical analysis. 176-180. I suggest to rephrase the sentence to make results more understandable. Basically in each group (prednisolone at 10 mg, 15 mg, or 20 mg) each parameter was analysed for differences across time (one-week post-treatment minus baseline- Δ), then a comparison between the results obtained with different doses was performed. In my opinion the idea of time*dose interaction is misleading.  Did you try to compare baseline and FU parameters by a paired sample T test? Did you notice any difference? | *We thank the reviewer for this insightful and constructive comment. As suggested, we conducted paired sample t-tests (or Wilcoxon signed-rank tests, as appropriate) within each dose group to assess within-subject changes from baseline to post-treatment. These analyses confirmed the direction and statistical significance of changes observed with our main model. However, we still believe that, compared with paired sample t-test, the two-way repeated measures ANOVA approach allows for simultaneous evaluation of within-subject changes over time and between-group differences, while crucially accounting for baseline imbalances across dose groups, an important consideration given our modest and unequal sample sizes. We have now amended the methods section to better explain our approach and improve readability of our results.* | Page 8,  Lines 181-185 |
| **3** | Abstract.  -Purpose. Please correct the sentence. There is limited data on dose-specific metabolic effects OF exogenous glucocorticoids (GC) doses. | *We are sorry for the typo, we have now amended the text accordingly.* | Page 3, line 33 |
| **4** | Abstract.  -Line 35. Since just one bone marker has been evaluated I suggest to modify bone health with bone metabolism. | *We agree with the reviewer, this has been changed accordingly throughout all the manuscript.* | Page 3, line 35  Page 6, line 111 |
| **5** | Abstract.  -Line 48. Please add the p value for osteocalcin comparisons. | *Done* | Page 3, line 48 |
| **6** | Line 110.Please clarify what M/I value means. | *Done* | Page 6, line 112-113 |
| **7** | Lines 191-192. The authors stated that blood pressure remained stable in all groups throughout the study time. If blood pressure was evaluated at baseline and at the end of the study I would say that blood pressure remained stable in all groups at the end of the study. | *Thanks for the suggestion, now incorporated into the manuscript.* | Page 9, lines 207-208 |
| **8** | Table 2 footnotes. Pleas add EGP and M/I | *Done* | Page 17, Table 2 footnotes |
| **Reviewer 2 general comment:** | | | |
| This is a well-executed, clearly written, and clinically relevant study addressing an important gap in the literature: the short-term, tissue-specific, and dose-dependent effects of low-to-intermediate doses of prednisolone in healthy individuals. By pooling data from three clinical trials, the authors present a compelling and mechanistically insightful comparison of 10, 15, and 20 mg daily doses of prednisolone.  Although the sample size (particularly for the 10 mg group) is limited, the integration of data from three tightly controlled trials using consistent methodologies, including low-dose hyperinsulinemic-euglycemic clamps to assess tissue-specific insulin sensitivity, remains a major strength. The study provides valuable insights beyond conventional clinical markers such as HOMA-IR.  The manuscript is well-structured and clearly presented. The clinical implications are thoughtfully articulated, especially in relation to dose titration and glucocorticoid risk stratification. The inclusion of bone metabolism markers, albeit in a subset, adds translational relevance.  I believe this manuscript merits publication, pending the following minor revisions and clarifications: | | | |
|  | **Reviewer’ specific comments** | **Author response and changes made** | **Page and line numbers in revised paper where the change can be found** |
| **1** | The 10 mg dose group includes only five participants, raising concerns regarding statistical power and robustness of intergroup comparisons. While this is noted in the limitations, it would benefit from clearer emphasis in the Discussion section, ensuring that conclusions are appropriately tempered and free of overstatement. | *We thank the reviewer for highlighting this important limitation. As correctly noted, the 10 mg dose group includes only five participants, and we fully acknowledge that this limits the statistical power and precision of comparisons involving this group. We have now expanded the Discussion section to emphasize this more clearly and to ensure that our conclusions remain appropriately cautious when interpreting dose-related effects, particularly in relation to the 10 mg group. In particular, we have avoided any overstatement regarding the absence of changes at this dose and now frame this as a finding that requires further validation in larger cohorts.* | Pages 12  Lines 339-342 |
| **2** | The data suggest a dose-responsive worsening of adipose tissue insulin resistance, with a plateau effect in hepatic insulin resistance. It would be valuable to explicitly highlight the clinical significance of these findings. If feasible, please report effect sizes (e.g., Cohen's d or partial η²) for changes in key variables (M/I, EGP, and NEFA) to help contextualize whether observed differences are modest or substantial in magnitude. | *We thank the reviewer for this excellent suggestion. We have now calculated and reported effect sizes for the main metabolic endpoints to better convey the magnitude and clinical relevance of the observed changes. Specifically, we report partial eta squared (η²) values for the repeated measures ANOVA models. These values have been added to the Results section and we have updated the Methods accordingly. The effect sizes support the interpretation that the observed changes are not only statistically significant but also of substantial magnitude. We believe this addition strengthens the translational impact of the findings.* | Pages 9 (Methods), lines 194-197  Pages 10-11 (Results)  Lines 225-307  Page 12, line 363 |
| **3** | In some sections, the term "dose-dependent" is used even when the dose*time interaction is not statistically significant (e.g., line 210). Consider rephrasing to reflect the presence of a trend without overstating statistical certainty. | *We thank the reviewer for this valuable comment. We have revised the relevant sentences to clarify the interpretation and avoid overstating statistical certainty.* | Pages 11-12 |
| **4** | Please ensure the following typographical errors are corrected  o Line 263: Remove the extraneous "for" in "ceiling effect for of prednisolone."  o Line 353: Please clarify the phrase "a modest increase of 5 or 10 mg" to avoid ambiguity | *We have now corrected the typo and revised the sentence to improve clarity.* | Page 11, line 329  Page 15, lines 430-431 |
| **5** | Please ensure that Figure 2 clearly labels the individual tissue domains (a-d), with corresponding descriptors in the figure legend for clarity. | *We thank the reviewer for this helpful suggestion. As recommended, we have revised Figure 2 to ensure that each tissue domain is clearly labeled (panels a–g) and have updated the corresponding figure legend to explicitly associate each panel with its respective physiological domain.* | Page 19,  Lines 466-469  Figure 2 |
| **6** | In Table 2, define the delta symbol (Δ) in the legend to ensure clarity for readers unfamiliar with the shorthand. | *Done* | Page 17,  Lines 460 |
